# Incidence of atrial fibrillation after coronary artery bypass graft surgery and percutaneous coronary intervention: a prospective 2-year follow-up observational study

**DOI:** 10.1136/bmjopen-2025-106364

**Published:** 2025-11-28

**Authors:** Anders Wickbom, Espen Fengsrud, Joakim Alfredsson, Johan Engdahl, Torbjörn Kalm, Anders Ahlsson

**Affiliations:** 1 Department of Cardiothoracic and Vascular Surgery, Örebro University Hospital, Örebro, Sweden; 2 Department of Medical Sciences, Örebro University, Örebro, Sweden; 3 Department of Cardiology, Örebro University Hospital, Örebro, Sweden; 4 Division of Cardiology, Linköping University, Linköping, Sweden; 5 Department of Health, Medicine, and Caring Sciences, Linköping University, Linköping, Sweden; 6 Department of Clinical Sciences, Karolinska Institutet, Stockholm, Sweden; 7 Department of Cardiology, Danderyd Hospital, Stockholm, Sweden

**Keywords:** Adult cardiology, Coronary heart disease, Coronary intervention, Ischaemic heart disease, Cardiac surgery

## Abstract

**Background:**

Atrial fibrillation is a common arrhythmia in patients with ischaemic heart disease. New-onset atrial fibrillation after coronary revascularisation is associated with adverse cardiovascular outcomes. This study aimed to determine the long-term cumulative incidence of new-onset atrial fibrillation after percutaneous coronary intervention or coronary artery bypass grafting surgery.

**Methods:**

A prospective observational cohort study in a real-world population setting, conducted at three tertiary centres, on new-onset atrial fibrillation incidence after percutaneous coronary intervention (N=123) or coronary artery bypass grafting (N=123). Heart rhythm was monitored the first 30 days in hospital by telemetry and on discharge using a handheld thumb ECG device three times a day, and thereafter for 2-week periods at 3, 12 and 24 months. The primary endpoint was the cumulative incidence of new-onset atrial fibrillation 24 months after the index procedure. Secondary objectives were to describe the incidence of cerebral ischaemic stroke and bleeding, myocardial infarction and major bleeding events during 24 months follow-up.

**Results:**

Mean age was 67 years, and male sex was more prevalent. At 30 days, the cumulative incidence of atrial fibrillation was 56% (69/123) in the coronary artery bypass graft group and 2% (3/123) in the percutaneous coronary intervention group. At 24 months, the cumulative incidence of atrial fibrillation was 58% (71/123) in the coronary artery bypass graft group and 6% (7/123) in the percutaneous coronary intervention group. Stroke, myocardial infarction and major bleeding were infrequent during follow-up.

**Conclusion:**

Over 24 months of follow-up, incident new-onset atrial fibrillation mainly occurred during the first 30 days after coronary artery bypass grafting but was more evenly distributed during 24 months after percutaneous coronary intervention.

**Trial registration number:**

NCT04307225.

**STRENGTHS AND LIMITATIONS OF THIS STUDY**

- This was a multicentre true prospective observational study investigating the 2-year cumulative incidence of new-onset atrial fibrillation after an index cardiac revascularisation procedure for non-emergency ischaemic heart disease.
- A systematic and prospective arrhythmia monitoring strategy over 2 years was used on a real-world population of patients with no prior history of atrial fibrillation undergoing either coronary artery bypass grafting or percutaneous coronary intervention.
- Heart rhythm was monitored in-hospital with telemetry and after discharge intermittently three times a day with a hand-held thumb-ECG arrhythmia monitoring device.
- The non-randomised study design provides baseline differences, which reflect the current guidelines for the choice of revascularisation strategy, thus prohibiting any causal inference or comparison between the groups.

## Introduction

Over the last 30 years, cardiac revascularisation via coronary artery bypass graft (CABG) has decreased in favour of percutaneous coronary intervention (PCI), currently the most common revascularisation strategy for coronary artery disease.^1 2^ While new-onset atrial fibrillation (AF) has been reported in 18%–31% of patients after CABG^3^,^5^ and 0.1%–6% of patients after PCI,^6^,^10^ data are limited to retrospective in-hospital AF monitoring, where data on preoperative AF status is not always available.

Several studies have demonstrated an association between new-onset AF and increased long-term mortality^34 11^,^14^ after CABG. In the post hoc analysis of the EXCEL (Evaluation of XIENCE vs Coronary Artery Bypass Surgery for Effectiveness of Left Main Revascularisation) trial, which compared CABG to PCI for left main stem coronary disease, new-onset AF was an independent predictor of stroke and death after 3 years.^6^ Importantly, AF developed in 0.1% of patients after PCI compared with 18% of patients after CABG. Other prospective trials comparing CABG and PCI have consistently shown a significantly higher long-term stroke incidence after CABG,^15 16^ where subclinical AF could be a contributing factor. However, to our knowledge, no prospective study has investigated the long-term post-procedure AF incidence in these groups.

The prospective observational AFAF (Atrial Fibrillation After CABG and PCI) study is investigating the incidence of new-onset AF on a real-world population of patients undergoing isolated CABG or PCI for non-emergency ischaemic heart disease during a 24-month follow-up period. In our previously published 30-day follow-up, the cumulative incidence of new-onset AF was 56% after CABG and 2% after PCI, with a strong predictive association between CABG and new-onset AF. Moreover, new-onset AF occurred not only during hospitalisation but also after discharge.^17^ In this study, we present the results for the entire 24-month follow-up period.

## Methods

The AFAF study has been previously described.^17^ Briefly, patients with no prior history of AF, undergoing standard care non-emergency isolated CABG or PCI, were monitored for AF after their index procedure, with in-hospital telemetry and post-discharge monitoring with a Zenicor II device (Zenicor Medical Systems AB, Stockholm, Sweden). The inclusion criteria were isolated coronary intervention due to stable/unstable angina or non-ST-elevation myocardial infarction. The exclusion criteria were previously documented AF; bleeding disorder contraindicating oral anticoagulant (OAC) treatment; severe cognitive impairment; life expectancy <1 year; ongoing OAC treatment for any reason; for patients undergoing CABG, PCI within 3 months of their procedure; and for patients undergoing PCI, CABG within 3 months of their procedure.

**Figure 1:**
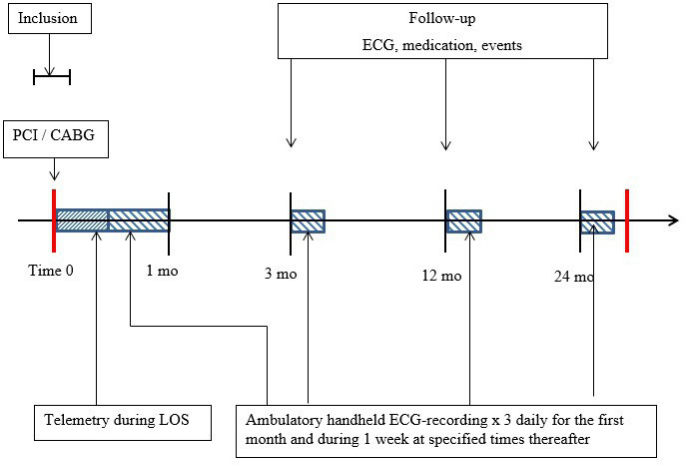
Study timeline. CABG, coronary artery bypass graft; LOS, length of stay; PCI, percutaneous coronary intervention.

The AFAF study was conducted at three Swedish centres: Örebro University Hospital, Linköping University Hospital and Danderyd Hospital (Stockholm). Patient enrolment started in May 2016 and ended in April 2019. It followed the principles of the Declaration of Helsinki,^18^ was approved by the regional ethics committee in Uppsala, Sweden (2015/413) and was registered at ClinicalTrials.gov (NCT04307225). Written informed consent was obtained from all participants. An external medical committee was appointed to evaluate adverse events and protocol changes. Consecutive eligible patients were asked to participate during their hospital stay for the coronary intervention between 1 day before and 5 days after the index procedure (figure 1). CABG patients underwent standard isolated on-pump (perioperative cardiopulmonary by-pass) revascularisation surgery through a midline sternotomy approach. Graft vessels included the left internal mammary artery and the saphenous vein used at the discretion of the individual surgeon. PCI patients underwent a standard treatment procedure with arterial endovascular access through the right radial artery and, if not accessible, the left radial or right femoral artery. Data on graft usage in the CABG groups and the distribution of stent types in the PCI group is provided in online supplemental material 1.

### Arrhythmia monitoring and follow-up

New-onset AF was defined as an episode of irregular R-R intervals and the absence of P waves lasting >30 s. The Zenicor II device uploads one-lead 30 s ECG strip data to a web-based, password-protected, study-specific database and uses a built-in arrhythmia detection algorithm. It has been thoroughly validated as a reliable AF screening device^19^,^22^ and the built-in algorithm was validated for AF detection specifically, on the present study’s Zenicor II device recordings.^22^ A Zenicor II device was mailed to the study participants at 3, 12 and 24 months post-procedure, and they were asked to record an ECG three times daily for 2 weeks, after which they were contacted by telephone for a prespecified follow-up. All Zenicor II ECG recordings were monitored by study coordinators and study cardiologists (EF, JE, TK and JA). Any arrhythmia requiring intervention was reported to the patient and their local healthcare professional for further care. In the case of mortality, information on the cause of death was collected from the medical records, if possible. Thromboembolic and haemorrhagic data were collected from medical records and patient follow-ups. Major bleeding was defined as bleeding requiring surgical intervention, hospitalisation and/or blood transfusion. Minor bleeding was defined as bleeding requiring limited intervention, such as discontinuing antithrombotic medication, or outpatient treatment, such as cauterisation for nosebleeds.

### Antithrombotic therapy

All patients received standard of care antithrombotic therapy according to the European Society of Cardiology guidelines.^23 24^ After CABG, antiplatelet therapy with aspirin was initiated on the day of surgery. Patients were also routinely administered low-molecular-weight heparin for at least 5 days postoperatively. For patients with acute coronary syndrome or drug-eluting stents, dual antiplatelet therapy (DAPT; aspirin with clopidogrel/prasugrel/ticagrelor) was considered postoperatively. After PCI, patients were routinely administered DAPT. After both CABG and PCI, if the patient developed AF, their stroke risk was estimated using the Congestive heart failure, Hypertension, Age (≥75 years), Diabetes, Stroke (or TIA), Vascular disease, Age (65-74 years), and Sex category (female) (CHA_2_DS_2_-VASc) score and their individual bleeding risk was assessed; if their CHA_2_DS_2_-VASc score was ≥2, OAC with warfarin or direct oral anticoagulants was initiated unless contraindicated. For the following 24 months, antiplatelet and/or OAC treatment was initiated or discontinued at the discretion of the patient’s treating physician.

### Statistical analysis

Based on retrospective data and assuming an increasing incidence of AF over time (by detection of subclinical AF), we estimated a cumulative incidence of AF of 35% in the CABG group and 15% in the PCI group over the 24-month follow-up. Considering an estimated power of 90% and a significance level of 5%, 194 participants were required. To allow for missing data and a 15% patient drop-out during follow-up, 250 participants were required.

Categorical and continuous variables were calculated descriptively and presented as the mean±SD, percentage (number) or median (IQR). The cumulative AF incidence over time was illustrated with the Kaplan-Meier method. All statistical analyses were performed using SPSS (V.22 and 27; IBM, Armonk, New York, USA).

### Patient and public involvement statement

Patients and the public were not involved in the design, conduct, reporting or dissemination plans of our research. Patients were involved informally when first receiving the study information in writing and verbally by study personnel, and formally on written informed consent. The patients and public were not involved in choosing the outcome measures as they were based on previous research in the field. Information on the study, its time plan, funding and progress was available to the public through registration through the public records of the regional ethics committee in Uppsala, Sweden (2015/413), register at ClinicalTrials.gov (NCT04307225), the main authors affiliated department regional public registries for clinical trials and the applications of funding.

## Results

**Figure 2:**
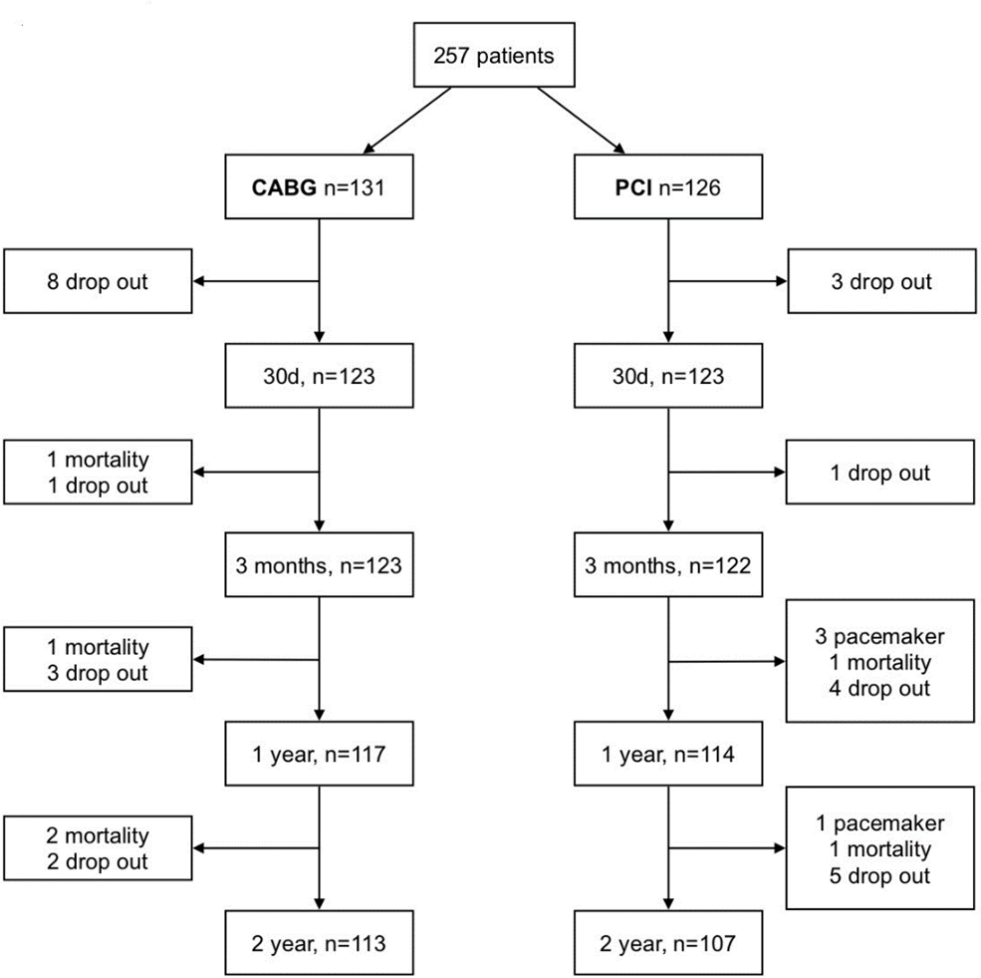
Consort diagram. CABG, coronary artery bypass graft; PCI, percutaneous coronary intervention.

The AFAF study included 257 patients, of whom 131 underwent CABG and 126 underwent PCI. At the end of the 24-month follow-up, participation had been discontinued in 37 patients, equally distributed between the two study arms (figure 2), of which 6 were due to death. At 3 months, one patient in the CABG group had died from myocardial infarction. At 12 months, two additional patients had died: one in the CABG group from septic shock and one in the PCI group from malignancy. At 24 months, three additional patients had died: two in the CABG group—one from malignancy and one from an unknown cause—and one in the PCI group from an unknown cause.

### Baseline characteristics

Mean age was 67±8 years in the CABG group and 67±9 years in the PCI group. More participants were male in the CABG group than in the PCI group (91% (112/123) vs 71% (88/123)). Diabetes mellitus (34% (40/119) vs 17% (21/122)), heart failure (13% (116/120) vs 2% (3/123)) and three-vessel disease (81% (100/123) vs 3% (4/123)) were more common in the CABG group than in the PCI group. Further, chronic obstructive pulmonary disease was more common in the PCI group, and peripheral vascular disease was more common in the CABG group (table 1).^17^ Nearly all patients in the PCI group (98% (120/123)) were discharged with DAPT*,* whereas 46% (28/60) of patients in the CABG group were discharged with OAC+aspirin.^17^ Data on angina status and revascularisation strategy is provided in online supplemental material 1, data on preoperative cardiovascular and antithrombotic drugs is provided in online supplemental material 2.

**Table 1:** Baseline characteristics

	CABG (n=123)	PCI (n=123)
Age, mean±SD	67±8	67±9
Female sex, % (n)	9 (11/123)	29 (35/123)
BMI (kg/m^2^), mean±SD	29±5	28±4
People who smoke % (n)	8 (10/119)	11 (14/123)
EtOH>8 standard drinks/week	6 (7/118)	6 (7/119)
Congestive heart failure, % (n)	13 (16/120)	2 (3/123)
Previous MI, % (n)	43 (41/120)	40 (49/123)
COPD, % (n)	1 (1/120)	4 (5/123)
OSA, % (n)	5 (6/120)	3 (4/123)
Hypertension, % (n)	68 (82/120)	67 (82/123)
Diabetes mellitus, % (n)	34 (40/119)	17 (21/122)
Previous stroke/TIA, % (n)	6 (7/119)	5 (6/123)
Peripheral vascular disease, % (n)	6 (7/120)	2 (3/123)
CHA_2_DS_2_-VASc, median (IQR)	3 (2)	3 (2)
Number of diseased coronary vessels, % (n)		
1	3 (4/123)	62 (76/123)
2	15 (19/123)	35 (43/123)
3	81 (100/123)	3 (4/123)
LA area (cm^2^), mean±SD	21±5	21±5
LVEF (%), mean±SD	55±11	57±8

Data are presented as the mean±SD, percentage (number *(*n)) or median (IQR).

BMI, body mass index; CABG, coronary artery bypass grafting; CHA2DS2-VASc, Congestive heart failure, Hypertension, Age (≥75 years), Diabetes, Stroke (or TIA), Vascular disease, Age (65-74 years), and Sex category (female); COPD, chronic obstructive pulmonary disease; DOAC, direct oral anticoagulants; LA, Left atrium; LVEF, left ventricular ejection fraction; MI, myocardial infarction; NSTEMI, non-ST-elevation myocardial infarction; OSA, obstructive sleep apnoea; PCI, percutaneous coronary intervention; TIA, transient ischaemic attack.

### New-onset AF, cumulative AF incidence and AF prevalence

**Figure 3:**
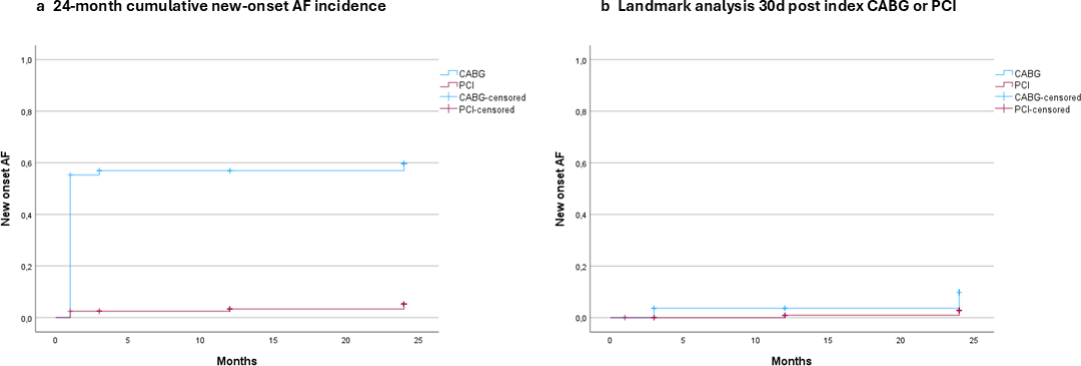
(a) 24-month cumulative new-onset AF incidence. (b) Landmark analysis 30 days post index procedure. AF, atrial fibrillation; CABG, coronary artery bypass graft; PCI, percutaneous coronary intervention.

At 30 days, the cumulative incidence of AF was 56% (69/123) in the CABG group and 2% (3/123) in the PCI group.^17^ At 3 months, one patient in the CABG group had developed new-onset AF. At 12 months, one patient in the PCI group had developed new-onset AF. At 24 months, one additional patient in the CABG group and four additional patients in the PCI group had developed new-onset AF, yielding a cumulative AF incidence of 58% (71/123) in the CABG group and 6% (7/123) in the PCI group (figure 3). To illustrate the difference in pre and post 30-day cumulative incidence rate of new-onset AF, a landmark analysis on patients unaffected by AF at 30 days after their index procedure was included (figure 3).

At 24 months, the prevalence of non-paroxysmal AF was 2% (2/113) in the CABG group and 2% (2/107) in the PCI group. The proportion of patients who experienced one or more AF episodes during the 2-week monitoring periods was 3% (4/121) in the CABG group and 1% (1/121) in the PCI group at 3 months, 4% (5/117) in the CABG group and 1% (1/114) in the PCI group at 12 months and 5% (6/113) in the CABG group and 5% (5/107) in the PCI group at 24 months (table 2).

**Table 2:** 3 months, 12 months and 24 months follow-up

3 months	CABG (n=121)	PCI (n=122)
AF at f-u*	2 (2/121)	0 (0/122)
SR at f-u	98 (119/121)	100 (122/122)
Any AF during f-u†	3 (4/121)	1 (1/122)
Aspirin	80 (96/121)	97 (118/122)
Clopidogrel	6 (7/121)	42 (51/122)
Ticagrelor	12 (14/121)	47 (57/122)
OAC‡	53 (64/121)	3 (4/122)
Thromboembolic event	1 (1/121)	1 (1/122)
Pericardial effusion	0 (0/121)	2 (2/122)
Cerebral haemorrhage	0 (0/121)	2 (2/122)
Cardiac ischaemia	2 (2/121)	2 (2/122)
Heart failure	1 (1/121)	2 (2/122)
Major bleeding§	3 (3/121)	2 (2/122)
Minor bleeding	2 (2/121)	3 (4/122)

12 months	CABG (n=117)	PCI (n=114)
AF at f-u*	2 (2/117)	0 (0/114)
SR at f-u	98 (115/117)	100 (114/114)
Any AF during f-u†	4 (5/117)	1 (1/114)
Aspirin	59 (69/117)	96 (109/114)
Clopidogrel	4 (5/117)	4 (5/114)
Ticagrelor	3 (3/117)	9 (10/114)
Any OAC‡	56 (65/117)	3 (4/114)
Thromboembolic event	0 (0/117)	0 (0/114)
Pericardial effusion	0 (0/117)	0 (0/114)
Cerebral haemorrhage	1 (1/117)	1 (1/114)
Cardiac ischaemia	3 (4/117)	3 (3/114)
Heart failure	1 (1/117)	0 (0/114)
Major bleeding§	3 (3/117)	3 (3/114)
Minor bleeding	7 (8/117)	1 (1/114)

24 months	CABG (n=113)	PCI (n=107)
AF at f-u*	2 (2/113)	2 (2/107)
SR at f-u	98 (111/113)	98 (105/107)
Any AF during f-u†	5 (6/113)	5 (5/107)
Aspirin	49 (55/113)	97 (102/107)
Clopidogrel	2 (2/113)	0 (0/107)
Ticagrelor	0 (0/113)	4 (4/107)
Any OAC‡	60 (68/113)	5 (5/105)
Thromboembolic event	1 (1/113)	1 (1/104)
Pericardial effusion	0 (0/113)	1 (1/104)
Cerebral haemorrhage	1 (1/113)	0 (0/104)
Cardiac ischaemia	2 (2/113)	2 (4/104)
Heart failure	0 (0/113)	0 (0/104)
Major bleeding§	1 (1/113)	2 (2/104)
Minor bleeding	3 (3/113)	2 (2/104)

Data are presented as % (n).

Missing data was limited to drug prescriptions (n=2) and complications (n=3) in the PCI group at the 24-month follow-up.

* Non-paroxysmal AF throughout the follow-up period.

† Includes ‘AF at f-u’.

‡ For data on DOAC/warfarin/LMWH see online supplemental material 3.

§ Includes cerebral haemorrhage.

AF, atrial fibrillation; CABG, coronary artery bypass graft; DOAC, direct oral anticoagulants; f-u, follow up; LMWH, low-molecular-weight heparin ; OAC, oral anticoagulation; PCI, percutaneous coronary intervention; SR, sinus rhythm.

### Complications, platelet inhibitors and OAC

Thromboembolic events (stroke and pulmonary embolism) were rare/infrequent during follow-up and at most 1% (table 2). Cardiac ischaemia had been reported in 2% (2/121) of patients in the CABG group and 2% (2/122) of patients in the PCI group at 3 months, 3% (4/117) of patients in the CABG and 3% (3/114) of patients in the PCI group at 12 months and 2% (2/113) of patients in the CABG group and 2% (2/104) of patients in the PCI group at 24 months (table 2). Major bleeding (intracranial or gastrointestinal bleeding) was reported in 2% (3/121) of patients in the CABG group and 2% (2/122) of patients in the PCI group at 3 months, 3% (3/117) of patients in the CABG group and 3% (3/114) of patients in the PCI group at 12 months and 1% (1/113) of patients in the CABG group and 2% (2/104) of patients in the PCI group at 24 months. Aspirin use remained consistent over time in the PCI group (97% (118/122) at 3 months, 96% (109/114) at 12 months and 97% (102/107) at 24 months). In contrast, aspirin use declined over time in the CABG group (80% (96/121) at 3 months, 59% (69/117) at 12 months and 49% (55/113) at 24 months). However, OAC use increased over time in the CABG group (53% (64/121) at 3 months, 56% (65/117) at 12 months and 60% (68/113) at 24 months; table 2). For detailed information on postoperative cardiovascular medication and OAC/direct oral anticoagulants drug types, see online supplemental material 3.

## Discussion

The main finding of our study is that the cumulative incidence of new-onset AF over the 24-month follow-up was 58% in the CABG group and 6% in the PCI group, using additional intermittent ECG monitoring. The incidence of new-onset AF after CABG in our study is higher than in previous prospective studies, which reported incidences of 16.8%–18.0%,^6 25^ and marginally higher than in previous retrospective studies, which reported incidences of 20%–40%.^3 26 27^ The incidence of new-onset AF after PCI in our study is consistent with previous studies, which reported incidences of 5%–6%.^7^,^10^

The second finding of our study is that cases of new-onset AF after CABG were concentrated in the first 30 days, whereas most cases of new-onset AF after PCI occurred later (12–24 months). To our knowledge, one previous study investigated the incidence of AF after CABG and PCI based on randomised data.^6^ While our study found a significantly higher incidence of AF after both CABG and PCI than Kosmidou *et al*,^6^ the difference between groups is similar. The prevalence of AF is 7.1%–7.9% in the general European adult population, with its incidence increasing with age.^28^ AF was not only significantly less common after PCI than CABG but also mainly developed late after the index procedure (12–24 months). Therefore, our finding of a cumulative incidence of 6% after 2 years and a prevalence of 5% during follow-up in the PCI group may reflect a baseline incidence of AF in the studied age groups. In contrast, AF mainly developed after CABG during the first 30 days after the index procedure, with most cases detected in-hospital by telemetry before discharge. In our previous study examining the first 30 days of follow-up, the procedure (CABG vs PCI) was a significant predictor for new-onset AF.^17^

The baseline differences in our non-randomised study reflect the current guidelines for the choice of revascularisation strategy,^24^ as such any inferred causality can only be considered hypothesis generating. Examining the baseline characteristics; three-vessel disease, diabetes mellitus and heart failure were more common among patients undergoing CABG, all of which are known risk factors for AF^29^ and thus could be a contributing factor to the higher incidence of AF after CABG than PCI. However, the inflammatory response differs substantially between CABG and PCI,^30 31^ with major surgery inducing a stronger inflammatory response than endovascular surgery. Inflammation is known to induce AF,^32^,^34^ and recent retrospective matched data support an association between increased postoperative inflammatory serum markers (C-reactive protein) and new-onset AF after CABG.^30^ In our study, most participants were treated for stable coronary disease and thus had no recent ischaemic episode. The significant difference in AF incidence between groups and our previous finding of a strong predictive association between the procedure (CABG) and new-onset AF^17^ suggests that AF after revascularisation for ischaemic heart disease could be caused by the surgical trauma rather than the underlying ischaemia.

Previous retrospective data on patients after CABG suggest an association between postoperative AF and worse long-term outcomes than those remaining in sinus rhythm (SR),^13^ with a twofold increased risk of cardiovascular and cerebral mortality and a sixfold increased risk of future AF.^3 4 11 12 35^ Taha *et al* found that new-onset postoperative AF was associated with a fourfold increased risk of future AF and an increased long-term risk of heart failure and thromboembolic events.^13^ The randomised EXCEL trial, comparing CABG to PCI for left main coronary disease, reported that a post-procedural AF episode was an independent predictor of stroke and death after 3 years.^6^ Other prospective trials comparing CABG to PCI have reported significantly higher incidences of stroke after CABG that increase over time.^15 16^ In contrast, one study found that after CABG, the risk of thromboembolic complications was lower among patients with new-onset AF than those with non-valvular AF and did not differ significantly from those who remained in SR after CABG.^14^

Our study could not confirm the increased morbidity of patients who developed AF demonstrated in the previous studies. In our study, the prevalence of OAC use in the CABG group was 60% after 2 years, reflecting the cumulative incidence of AF. In addition, our follow-up period was 24 months compared with 12–36 months in previous studies.^6 15 16^ Importantly, our study was not designed to investigate the preventive effect of OACs in patients with new-onset AF after CABG; therefore, its findings should not be regarded as any evidence in that direction. A randomised trial is currently ongoing (ClinicalTrials.gov: NCT04045665), and current guidelines support OAC use for new-onset AF after CABG (Class IIA, Level of Evidence B).^29^

An association exists between increased AF burden and increased risk of heart failure and worse cardiac and cerebrovascular survival.^29 36^ Our study conducted intermittent screening for subclinical AF using handheld ambulatory ECG recorders, which has proven an effective, non-invasive way to capture AF episodes that is superior to a 24-hour Holter monitor.^19^,^21^ In addition, a substudy within the AFAF study in which 40 patients undergoing CABG were monitored using an implantable loop recorder (Reveal LINQ; Medtronic, Minneapolis, Minnesota, USA) in addition to their intermittent monitoring reported that AF burden was 0.1% for those in paroxysmal AF at 12 months.^37^ This finding would indicate that while the cumulative incidence of new-onset AF 24 months after CABG was high in our study, the AF burden may be low. A recently published international large-scale survey showed that AF monitoring and detection strategies after CABG were heterogeneous during hospitalisation (telemetry) but unstructured (non-AF targeted) after discharge (standard ECGs during planned postoperative outpatient visits).^38^ Our results suggest that monitoring for AF during the first month postoperative after CABG could be reasonable. Technical advances over the last years provide a plethora of reliable non-invasive alternatives for outpatient monitoring, and algorithm-based arrhythmia detection simplifies diagnostics.^39^

Our study had several limitations. First, its non-randomised design led to baseline differences, which could have led to a higher incidence of AF in the CABG group, although mean age and left ventricular ejection fraction (both risk factors for incident AF) did not differ significantly. Furthermore, the observational design prohibits the possibility for reliable comparison between the groups, as such, any inferred differences between CABG and PCI in this setting are to be interpreted as hypothetical. Second, previous studies on the incidence of new-onset AF after PCI relied mainly on data for patients with ST-elevation myocardial infarction (STEMI).^7^,^10^ In contrast, our study included all patients except those with STEMI. Since STEMI is the indication for PCI in roughly 20% of cases,^1^ our results cannot be generalised to the entire PCI population. The observed incidence of AF after PCI was lower than anticipated, statistically suggesting that the PCI group may be underpowered. The relatively low number of participants, as well as the non-randomised study design, prohibits comparative analyses on outcome measures. Finally, gender distribution was skewed in the CABG group compared with the general Swedish CABG population (9% vs 19% females),^26^ which could influence the generalisability of our findings.

## Conclusion

In our study, the 24-month cumulative incidence of new-onset AF was 58% after CABG and 6% after PCI using intermittent ECG monitoring. Most cases of AF occurred during the first 30 days after CABG, whereas they were more evenly distributed over the follow-up period after PCI.

## Supplementary material

10.1136/bmjopen-2025-106364 online supplemental file 1

## Data Availability

Data are available upon reasonable request.
