# Supplementary material for: Incidence of atrial fibrillation after coronary artery bypass graft surgery and percutaneous coronary intervention: a prospective 2-year follow-up observational study
Source: BMJ Open. 2025 Nov 28;15(11):e106364. doi: 10.1136/bmjopen-2025-106364 (PMC12666116; doi:10.1136/bmjopen-2025-106364)
Supplement: online supplemental file 1 [file bmjopen-15-11-s001.docx]

**Supplementary material 1.** Baseline angina and revascularization data.

| **CABG (n=123)** |  |
| --- | --- |
| Number of peripheral anastomoses | 3.4 ± 1.0 |
| LIMA utilization | 90% (110/123) |
| Angina, % (*n*) |  |
| Unstable | 21% (26/123) |
| Stable | 50% (62/123) |
| NSTEMI | 29% (35/123) |
| **PCI (n=123)** |  |
| Number of treated segments | 1.6 ± 0.9 |
| DES | 97% (119/123) |
| BMS | 1% (1/123) |
| Angina, % (*n*) |  |
| Unstable | 14% (17/123) |
| Stable | 54% (66/123) |
| NSTEMI | 33% (40/123) |

Data is presented as mean ± SD, or %(n). CABG = coronary artery bypass grafting, LIMA = left internal mammary artery, PCI = percutaneous coronary intervention, DES = drug eluting stent, BMS = bare metal stent

**Supplementary material 2.** Preoperative medication

| **Drug** | **CABG n** | **PCI** |
| --- | --- | --- |
| Aspirin | 90% (111/123) | 87% (107/123) |
| Clopidogrel | 2% (2/123) | 32% (39/122) |
| Ticagrelor | 4% (5/123) | 38% (46/123) |
| Beta blockers | 74% (91/123) | 50% (61/123) |
| ACEinh/ARB | 66% (81/123) | 55% (68/123) |
| MRA | 7% (8/123) | 1% (1/122) |
| Statins | 85% (105/123) | 73% (90/123) |
| DOAC | 0 | 0 |
| Warfarin | 0 | 0 |
| LMWH | 9% (11/123) | 2% (2/123) |

CABG = coronary artery bypass grafting, PCI = percutaneous coronary intervention, ACEinh/ARB = angiotensin converting enzyme inhibitor/angiotensine II receptor block, MRA = mineralocorticoid receptor antagonist, DOAC = direct oral anticoagulant, LMWH = low molecular weight heparin

**Supplementary material 3. Postoperative medication**

| **3-month** | **CABG (*n* = 121)** | **PCI (*n* = 122)** |
| --- | --- | --- |
| Beta-blockers | 94% (114/121) | 69% (84/122) |
| ACE/ARB | 77% (92/121) | 74% (90/122) |
| MRA | 8% (10/121) | 1% (1/122) |
| Statins | 95% (115/121) | 95% (116/122) |
| Dabigatran | 4% (5/121) | 1% (1/122) |
| Rivaroxaban | 4% (5/121) | 0% (0/122) |
| Apixaban | 26% (31/121) | 2% (2/122) |
| Edoxaban | 2% (2/121) | 0% (0/122) |
| Warfarin | 16% (19/121) | 1% (1/122) |
| LMWH | 2% (2/121) | 0% (0/122) |
| **12-months** | **CABG (*n* = 117)** | **PCI (*n* = 114)** |
| Beta-blockers | 91% (106/117) | 71% (81/114) |
| ACE/ARB | 76% (89/117) | 76% (87/114) |
| MRA | 8% (9/117) | 1% (1/114) |
| Statins | 90% (105/117) | 94% (107/114) |
| Dabigatran | 3% (4/117) | 1% (1/114) |
| Rivaroxaban | 7% (8/117) | 0% (0/114) |
| Apixaban | 33% (39/117) | 2% (2/114) |
| Edoxaban | 3% (3/117) | 0% (0/114) |
| Warfarin | 9% (11/117) | 1% (1/114) |
| LMWH | 0% (0/117) | 0% (0/114) |
| **24-months** | **CABG (*n* = 113)** | **PCI (*n* = 107)** |
| Beta-blockers | 89% (100/113) | 64% (67/105) |
| ACE/ARB | 75% (85/113) | 75% (79/105) |
| MRA | 14% (16/113) | 6% (6/105) |
| Statins | 90% (101/113) | 91% (96/105) |
| Dabigatran | 4% (5/113) | 0% (0/105) |
| Rivaroxaban | 9% (10/113) | 0% (0/105) |
| Apixaban | 36% (41/113) | 5% (5/105) |
| Edoxaban | 4% (4/113) | 0% (0/105) |
| Warfarin | 7% (8/113) | 0% (0/105) |
| LMWH | 0% (0/113) | 0% (0/105) |

Data are presented as % (n). ACE inh/ARB = Angiotensin-converting enzyme inhibitor/angiotensin receptor blocker, MRA = mineralocorticoid receptor antagonist, LMWH = low-molecular-weight heparin.

Missing data was limited to drug prescriptions (*n* = 2) in the PCI group at the 24-month follow-up
