# Supplementary material for: Incidence of atrial fibrillation after coronary artery bypass graft surgery and percutaneous coronary intervention: a prospective 2-year follow-up observational study
Source: BMJ Open. 2025 Nov 28;15(11):e106364. doi: 10.1136/bmjopen-2025-106364 (PMC12666116; doi:10.1136/bmjopen-2025-106364)
Supplement: Supplementary file 2 [file bmjopen-2025-106364.reviewer_comments.pdf]

PEER REVIEW HISTORY

BMJ Open publishes all reviews undertaken for accepted manuscripts. Reviewers are asked to complete a checklist review form and are provided with free text boxes to elaborate on their assessment. These free text comments are reproduced below.

ARTICLE DETAILS

Title (Provisional)

Incidence of Atrial Fibrillation After Coronary Artery Bypass Graft Surgery and Percutaneous Coronary Intervention: A prospective two-year follow-up study

Authors

Wickbom, Anders; Fengsrud, Espen; Alfredsson, Joakim; Engdahl, Johan; Kalm, Torbjörn; Ahlsson, Anders

---

VERSION 1 - REVIEW

Reviewer **1**

Name **Hueso, Miguel**

Affiliation **Universitari Bellvitge and Bellvitge Research Institute (IDIBELL), Nephrology**

Date **30-Jun-2025**

COI **None**

This is a descriptive study investigating the incidence of atrial fibrillation (AF) following coronary revascularization, either by percutaneous coronary intervention (PCI) or coronary artery bypass grafting (CABG), over a 24-month follow-up period. The authors reported a cumulative incidence of AF of 60% (74/123) in the CABG group and 7% (8/123) in the PCI group.

I have several concerns regarding the study:

1. The study lacks details about the CABG procedure. Important information, such as the duration of the surgery, the use of extracorporeal circulation, and whether any patients underwent concomitant valve surgery, is missing.

2. It is unclear whether there were any distinguishing clinical or demographic characteristics between patients who developed AF and those who did not. Additionally, no information is provided regarding preoperative echocardiographic findings.

3. As this is an observational study, the rationale for calculating a sample size (n) is questionable and may not be appropriate.

4. The list of treatments does not include diuretics, which are commonly prescribed in this patient population and could potentially influence the incidence of AF.

**Reviewer**	**2**
**Name**	**Sahin, Irfan**
**Affiliation**	**Istanbul Bagcilar Training and Research Hospital**
**Date**	**25-Jul-2025**
**COI**	**None**

This article presents 2-year extended follow-up data from a previous study. In this context, its specificity is limited. Also, the number of patients in the study is relatively small compared to other similar studies. It is recommended that the contribution of the 2-year follow-up data of the study to the current literature be better explained.

VERSION 1 - AUTHOR RESPONSE

Reviewer: 1
Dr. Miguel Hueso, Universitari Bellvitge and Bellvitge Research Institute (IDIBELL)
Comments to the Author:
This is a descriptive study investigating the incidence of atrial fibrillation (AF) following coronary revascularization, either by percutaneous coronary intervention (PCI) or coronary artery bypass grafting (CABG), over a 24-month follow-up period. The authors reported a cumulative incidence of AF of 60% (74/123) in the CABG group and 7% (8/123) in the PCI group.
I have several concerns regarding the study:
1. The study lacks details about the CABG procedure. Important information, such as the duration of the surgery, the use of extracorporeal circulation, and whether any patients underwent concomitant valve surgery, is missing.

Thank you for this remark. It is declared in free text form that the CABG group underwent isolated CABG surgery however we understand the rationale that the current presentation makes it less clear, all patients underwent CABG using cardiopulmonary by-pass but this was not disclosed. Unfortunately, information on duration of surgery is not available, we opted to not register this in the CRF as we empirically in our experience have found this to be a subjective measurement prone to registration bias.

We have edited some information to in better detail disclose perioperative surgical data in free text form. We have done the same for the PCI group to hopefully improve the description throughout. Further, some additional information in this matter has been addad as a supplemental material.

2. It is unclear whether there were any distinguishing clinical or demographic characteristics between patients who developed AF and those who did not. Additionally, no information is provided regarding preoperative echocardiographic findings.

Thank you. As the primary outcome in the PCI groups was low, and lower than expected, we thought that such an analysis would be inconclusive. One could argue that we could have done it for the CABG group where the outcome was higher, however, to be uniform in the presentation of data we chose not to do so as the added value would be hard to interpret in this context, and as the study was not designed for comparative analysis between the groups. It is however a valuable input and could be a intreaguing part of a post-hoc analysis in the CABG group alone.

Regarding preoperative echocardiographic findings we respectfully disagree. Data on left ventricular ejection fraction and left atrial area is provided at the end in table 1 (baseline characteristics) as this were the two (in our opinion) most valuable findings to present in this context.

3. As this is an observational study, the rationale for calculating a sample size (n) is questionable and may not be appropriate.

Thank you, yes this is somewhat correct. Although the study is observational and no intervention or treatment was given in context of the study, we still chose to perform a pre study sample size calculation as we wanted to be sure to have a sample size ample enough, ensuring that the primary outcome measure results were less prone to be a chance finding. Especially for the PCI group.

4. The list of treatments does not include diuretics, which are commonly prescribed in this patient population and could potentially influence the incidence of AF.

Thank you. We have revised the text in accordance with this remark and in conjunction to the remarks from the editor on clarifying that the patients underwent standard care. Implementing a supplementary material 2 showing preoperative medications, where mineral corticoid receptor antagonists are shown -as this is presented already in table 2. Long-term follow up. Data on MRAs were gathered and presented as it plays to the treatment or lack thereof on heart failure. However, loop diuretics, was administered at the discretion of each individual treating physician, and non-systematic. As such we did not register any in-hospital administration, nor post discharge prescription of diuretics. The CABG group most certainly all in some context received diuretics at least during postoperative ICU care, but as this is erratic and non-systematic, we found such data to be less valuable in this context.

Reviewer: 2
Dr. Irfan Sahin, Istanbul Bagcilar Training and Research Hospital
Comments to the Author:
This article presents 2-year extended follow-up data from a previous study. In this context, its specificity is limited. Also, the number of patients in the study is relatively small compared to other similar studies. It is recommended that the contribution of the 2-year follow-up data of the study to the current literature be better explained.

Thank you for your valuable input. It is true that the sample size is relatively small, especially in comparison to the post-hoc analysis of the EXCEL trial by Kosmidou et.al, referred to in the manuscript. We however believe that we have brought up the relevant literature on reports from

prospectively collected data, as well as retrospective cohort analyses and discussed our results in relation to this. As this is an addition of results from our previously published 30-day follow-up, the focus of the present paper was the long-term cumulative arrhythmia incidence and observationally describing adverse events. In our previous publication we performed a regression analysis, determining risk factors for incident AF. Such an analysis could have been an addition to this 24-month follow-up, but as the added outcome measure remained scarce in the timeframe from the previous publication (30-days follow-up) and the now presented 24-month data, it would not be of any significant contribution. As such we chose to focus on the cumulative incidence findings (in our opinion a valuable contribution since this was a true prospective follow-up with a systematic heart rhythm monitoring strategy), and discussing these to existing adjacent literature which to our best of knowledge does not, to any significant extent, include trials with a similar design as ours. As the secondary outcome measures was observed at low incidences, and given the observational design, any other outcome analysis – we thought – was not beneficial.
